# Plenary Symposium

**DOI:** 10.1111/ene.70233

**Published:** 2025-06-21

## Sunday, June 22 2025

## Presidential Symposium

## PLEN02_1

### How the Brain Represents the World

#### H. Sompolinsky^1,2^

##### ^1^Harvard University, Cambridge, USA; ^2^Hebrew University of Jerusalem, Jerusalem, Israel

One of the brain's most profound functions is to represent the world around us—transforming continuous, noisy streams of sensory input into stable internal models of the environment. These internal models support and guide perception, memory, cognition, and, most importantly, predictions about the state of the world. A leading hypothesis is that these internal representations correspond to stable patterns of activity in large populations of neurons. In this lecture, I will explain how such patterns emerge from the intrinsic dynamics and architecture of neuronal circuits. While the circuit dynamics constrain the repertoire of possible stable states, the specific state expressed at any given time is determined by ongoing sensory input. Disruptions in the balance between intrinsic dynamics and sensory‐driven responses may underlie a range of neurological and psychiatric disorders. I will illustrate this principle using neuronal circuit models for memory, spatial navigation, object recognition, and language processing. This theory is supported and enriched by advances in artificial deep neural networks and generative AI, which provide powerful tools for testing hypotheses about brain function at realistic scales and levels of complexity.

**Disclosure:** Nothing to disclose.

## PLEN02_3

### Multiple sclerosis, from biology to clinical translation. A focus on nodes of Ranvier and electrical activity

#### C. Lubetzki

##### Salpetriere Hospital, Paris, France

Multiple sclerosis (MS), an autoimmune demyelinating and degenerative disease of the CNS, remains only partly responsive to immunotherapies; despite reducing relapse rate, these immunomodulator/immunosuppressive drugs have not shown yet a convincing impact on disability progression. As remyelination might prevent axonal damage (although some controversies are ongoing…), finding new ways to promote remyelination and neuroprotection is now the next frontier for MS. Among innovative/disruptive perspectives from the recent years, we will here focus on the following steps necessary for CNS remyelination; and notably on the newly discovered neuron‐glial interactions at the node of Ranvier, between node and oligodendrocyte precursors on the one hand, between node and microglia on the other hand. These studies led to the identification of clusters of nodal proteins detected prior to myelin deposition, structures that we named “pre‐nodes”. These prenodes i) appear on axons prior to myelination onset, ii) participate to node of Ranvier formation, iii) accelerate propagation of axon electrical potential, leading to the new concept of increased conduction independent of myelin deposition, iv) are induced by a contactin‐secreted oligodendroglial complex. In addition to these oligodendrocyte‐node of Ranvier contacts, microglia have emerged as another major type of neuron glia interactions (for sake of time this field will be only alluded during the talk). Importantly, these interactions are both regulated by neuronal electrical activity, as shown using different techniques such as Dreadds and optogenetics. The established impact of electrical activity on (re)myelination prompted us to set up a small size study where trans‐orbital electrical stimulation was used to favor remyelination after an episode of optic neuritis (Dr Beigneux, Pr Louapre, Paris, ICM; collaborations Prs Leocani (Milan), Sahel, Vignal and Touati (Paris). This controlled, recently completed study shows a tendency to reduced VEP latency between “stimulated” and “sham” group. A study assessing MEG latency and harmonics (N. George; B. Rossion, Paris) was added. Altogether, despite limitations, (small size of the study, MEG markers still pending…), human translation has suggested that stimulation of electrical activity might favor remyelination in the optic nerve opening novel perspectives of treatment for MS progression.

**Disclosure:** Nothing to disclose.

## PLEN02_5

### Protein aggregation and its relevance for neurodegenerative diseases

#### M. Spillantini

##### Clifford Allbutt Building, Cambridge, UK

## PLEN02_7

### Sleep by the brain, for the brain: Implications for neurology

#### C. Bassetti

##### University of Bern, Inselspital, Switzerland

## PLEN02_9

### Myoclonus, you need to know it to see it

#### M. de Koning‐Tijssen

##### Department of Neurology, University Medical Center Groningen, Groningen, The Netherlands

Myoclonus is a hyperkinetic movement disorder characterized by brief, sudden, shock‐like jerks caused by hyperexcitable neurons, leading to either muscle activation (positive) or inhibition (negative). Myoclonus presents with a wide range of clinical manifestations and etiologies, including acquired causes such as medication side effects, metabolic or autoimmune conditions, and genetic disorders. This variability makes epidemiology unclear and contributes to under‐recognition in clinical practice. Distinguishing myoclonus from other movement disorders, such as tics, tremor, or dystonia, relies heavily on clinical examination. However, phenotyping remains challenging, even among experts, due to overlapping features and subjective interpretation. Electromyography (EMG) can provide objective diagnostic support, as different movement disorders exhibit distinct EMG patterns. However, diagnostic accuracy remains limited as sensitivity and specificity of most electrophysiological tests are lacking. New machine learning tools hold promises in supporting more accurate classification. A structured diagnostic approach that integrates clinical features with neurophysiological data, supported by the new classification system, can aid in localizing the anatomical origin of myoclonus, including cortical, subcortical, brainstem, spinal, or peripheral myoclonus. Cortical myoclonus is the most common subtype. Functional jerky movements are also frequent and should be considered during evaluation. Generally, acute onset with rapid progression suggests an acquired etiology, while early‐onset cases with slow progression point towards a genetic origin. Accurate identification of the underlying cause is important for guiding precision medicine‐based treatment strategies, that may differ markedly depending on the anatomical and etiological classification.

**Disclosure:** Nothing to disclose.

## Tuesday, June 24 2025

## Highlights and breaking news

## PLEN03_3

### Highlight: Dementia

#### S. Tomic

##### University Hospital Centre Osijek, Osijek, Croatia

## PLEN03_4

### Highlight: Headache

#### P. Pozo‐Rosich

##### Vall d'Hebron University Hospital, Barcelona, Spain

## PLEN03_5

### Highlight: Movement disorders

#### A. Tessitore

##### University of Campania, “Luigi Vanvitelli”, Naples, Italy

## PLEN03_6

### Highlight: Cerebrovascular diseases

#### T. Truelsen

##### Rigshospitalet, Copenhagen, Denmark

## PLEN03_7

### Highlight: Neuromuscular diseases

#### M. Filosto^1,2^

##### ^1^Department of Clinical and Experimental Sciences, University of Brescia, Brescia, Italy; ^2^NeMO‐Brescia Clinical Center for Neuromuscular Diseases, Brescia, Italy

Neuromuscular disorders represent an increasingly dynamic field with profound implications for clinical neurology. Recent advancements have significantly reshaped both diagnostic and therapeutic paradigms, encompassing the expanded application of muscle imaging techniques, omics‐based analyses, gene‐targeted therapies, and emerging immunotherapies. This presentation aims to provide neurologists with a focused update on critical advancements across motor neuron diseases, neuropathies, myopathies, and neuromuscular junction disorders, with a particular emphasis on translating these innovations into clinical practice to enhance diagnostic precision and optimize patient management

**Disclosure:** MF has served as a participant in advisory boards for Sanofi, Amicus, Johnson and Johnson, Zambon and Biogen.

## PLEN03_8

### Award Lecture

#### T. Emmenegger

##### Spinal Cord Injury Centre, Balgrist University Hospital, University of Zurich, Zurich, Switzerland

In spinal cord injury (SCI), magnetic resonance imaging (MRI) reveals tissue bridges and neurodegeneration for 2 years, while long‐term effects remained unknown. This study aims to track initial lesion changes, subsequent neurodegeneration, and their impact on recovery over 5 years. Twenty‐three acute SCI patients and 21 healthy controls were assessed clinically—and by MRI—regularly from 3 days up to 60 months post‐injury. We employed histologically cross‐validated quantitative MRI sequences sensitive to volume, myelin, and iron changes, reflecting indirect processes of neurodegeneration and neuroinflammation. General linear models tracked lesion and remote changes in volume, myelin‐and iron‐sensitive magnetic resonance indices over 5 years. Associations between lesion, degeneration, and recovery were assessed. Patients' motor scores improved by an average of 12.86 (95% confidence interval [CI]=6.70–19.00) points, and SCIM by 26.08 (95% CI=17.00–35.20) points. Within 3–28 days post‐SCI, lesion size decreased by more than two‐thirds (3 days: 302.52±185.80 mm^2^, 28 days: 76.77±88.62 mm^2^), revealing tissue bridges. Cervical cord and corticospinal tract volumes transiently increased in SCI patients by 5% and 3%, respectively, accompanied by cervical myelin decreases and iron increases. Over time, progressive atrophy was observed in both regions, which was linked to early lesion dynamics. Tissue bridges, reduced swelling, and myelin content decreases were predictive of long‐term motor score recovery and improved SCIM score. Studying acute changes and their impact on longer follow‐up provides insights into SCI trajectory, highlighting the importance of acute intervention while indicating the potential to influence outcomes in the later stages.

**Disclosure:** N.W. holds a patent on acquisition of MRI data during spoiler gradients (US 10,401,453 B2), which were parts of sequences used in this study. N.W. was a speaker at an event organized by Siemens Healthcare, which was the scanner brand used in this study, and was reimbursed for the travel expenses. The Max Planck Institute for Human Cognitive and Brain Sciences and Wellcome Centre for Human Neuroimaging have institutional research agreements with Siemens Healthcare, which as previously mentioned was the scanner brand used in this study.

